# Is size, morphology, site, and access scoring system consistent between endoscopists? Interobserver and intraobserver polyp assessment study

**DOI:** 10.1055/a-2752-2591

**Published:** 2025-12-09

**Authors:** Mo Thoufeeq, Ahmad Thaika, Shyam Moudhgalya, Pradeep Mundre, Vasitha Abeysuriya, Nilanga Nishad

**Affiliations:** 1 7318 Gastroenterology, Sheffield Teaching Hospitals NHS Foundation Trust, Sheffield, United Kingdom of Great Britain and Northern Ireland; 2 156766 FY2, Walsall Manor Hospital, Walsall, United Kingdom of Great Britain and Northern Ireland; 3 5983 Foundation Programme, Newcastle Upon Tyne Hospitals NHS Trust, Newcastle Upon Tyne, United Kingdom of Great Britain and Northern Ireland; 4 1906 Bradford Teaching Hospitals NHS Foundation Trust, Bradford, United Kingdom of Great Britain and Northern Ireland; 5 92955 Faculty of Medicine, University of Kelaniya, Kelaniya, Sri Lanka

**Keywords:** Endoscopy Lower GI Tract, Polyps / adenomas / ..., Endoscopy Upper GI Tract, Diagnosis and imaging (inc chromoendoscopy, NBI, iSCAN, FICE, CLE), Quality and logistical aspects, Performance and complications

## Abstract

**Background and study aims:**

This study aimed to evaluate interobserver and intraobserver agreement in Size/Morphology/Site/Access (SMSA) scoring among practicing endoscopists with varying levels of experience.

**Patients and methods:**

A total of 102 fully independent endoscopists participated in the study. Ten short video clips of colonic polyps of varying size and complexity were recorded using Olympus 290 colonoscopes and included white light, near focus, narrow band imaging, and chromoendoscopy where applicable. These videos were embedded in an online questionnaire. Endoscopists were asked to assign SMSA scores based on three variables—size, morphology, and access—with the site provided for standardization. A subgroup of five participants repeated the assessment after 2 to 3 weeks to evaluate intraobserver consistency. Data were analyzed using Fleiss’ kappa via SPSS (v26), and Kappa interpretation followed the Landis and Koch classification.

**Results:**

Overall interobserver agreement for SMSA level across all participants was fair (κ = 0.346). Among individual parameters, morphology had the highest agreement (κ = 0.505, moderate), followed by access (κ = 0.408, moderate) and size (κ = 0.241, fair). Subgroup analysis of experienced endoscopists (> 1000 lifetime colonoscopies) yielded slightly improved kappa values, with morphology still demonstrating the highest consistency. Intraobserver agreement showed moderate to almost perfect reliability for size (κ = 0.444 to 1.000) and moderate to substantial agreement for SMSA level (κ = 0.429 to 0.846).

**Conclusions:**

Morphology was the most consistently scored parameter. Although the SMSA tool remains valuable, efforts such as standardized training and simplification of variable categories may be warranted to improve interobserver consistency and enhance clinical-utility.

## Introduction

Colonoscopy remains the gold standard for colorectal cancer (CRC) screening. It provides diagnostic accuracy and therapeutic benefit by allowing removal of premalignant polyps, thereby reducing CRC incidence and mortality 1 . Although most polyps detected are small and straightforward to manage, a subset of lesions, particularly large non-pedunculated colorectal polyps (LNPCPs), pose technical challenges due to their size, morphology, and anatomical location 2 3 . Polyps exceeding 2 cm in size without a stalk are classified as LNPCPs, whereas “complex” non-pedunculated colorectal polyps (C-NPCPs) are defined by additional factors such as suspected malignancy, high recurrence risk, or elevated risk of incomplete resection or procedure complications 2 4 .

Effective management of these lesions relies on accuracy of pre-procedure characterization. The Size, Morphology, Site, and Access (SMSA) scoring system, introduced by Gupta et al. in 2011, was a major step forward in standardizing assessment of polyp complexity 5 . It offers a structured, additive scoring framework that predicts technical difficulty and potential complications during resection. This tool now informs credentialing standards, such as the Joint Advisory Group on Gastrointestinal Endoscopy colonoscopy certification, which requires proficiency in resecting SMSA level 2 lesions for independent practice 6 .

However, the SMSA system is not without limitations. Its four domains depend on visual and subjective estimation, which may lead to observer bias and variability, particularly among non-specialist or inexperienced endoscopists 7 8 . Interobserver variability has implications for patient referral accuracy, informed consent, and resource allocation in tertiary centers 9 10 . Despite its widespread clinical use, no study to date has comprehensively examined interobserver and intraobserver agreement in SMSA scoring using standardized video-based assessment across varying levels of expertise.

Given the emergence of artificial intelligence (AI) and computer-aided diagnosis (CADx) systems aimed at reducing interobserver variability 11 12 , reassessing reliability and reproducibility of the SMSA tool is particularly timely. This study sought to evaluate observer agreement in SMSA scoring, identify which domains are most prone to variability, and explore potential avenues for system refinement to enhance real-world utility.

## Material and methods

### Study design and polyp selection and video preparation

To assess consistency of SMSA scoring, our team of expert endoscopists (PM and MT), both experienced in resection of complex colonic polyps, recorded video clips of polyps with varying sizes and morphological complexities. These polyps were randomly selected from real clinical cases to represent a broad spectrum—from simple lesions to those typically requiring advanced endoscopic intervention. Videos were captured using Olympus 290 colonoscopes and processors, incorporating standard white-light imaging, near-focus, narrow band imaging, and, where necessary, chromoendoscopy with indigo carmine dye. Informed consent was obtained from all patients whose procedures were recorded. This was an exploratory observational study rather than a confirmatory trial. Primary and secondary outcomes were predefined for structured data analysis and interpretation, but the study was not designed or powered as a confirmatory trial according to CONSORT or other clinical trial guidelines.

This study was approved by the Sheffield Teaching Hospitals NHS Foundation Trust Research and Development Department. Ethical approval reference: STH21111. All procedures were conducted in accordance with institutional and national research ethics guidelines. Informed consent was obtained from all patients whose procedure videos were used for educational and research purposes.

### Questionnaire development

Each video was embedded into a structured online questionnaire. Participants were asked to assess three of the four SMSA variables—size, morphology, and access. The site parameter was predefined and provided with each video to reduce variability because it is considered an objective characteristic. The tool was designed to automatically calculate total SMSA score and assign the corresponding SMSA level based on participant input.

### Participant recruitment and grouping

A total of 102 independent colonoscopists were recruited to participate in the study. The majority (85/102) were based in the United Kingdom. The participant pool included consultant gastroenterologists, independent senior trainees, colorectal surgeons, and nurse endoscopists. No formal training on the SMSA tool was provided to participants prior to the assessment. Baseline demographic and professional data were collected, including the number of lifetime colonoscopies performed. Participants were classified as experienced (≥ 1000 lifetime colonoscopies; n = 72 and less experienced < 1000 lifetime colonoscopies; n = 30).

The study included endoscopists (n = 102) with a range of experience, drawn from multiple hospital settings (university hospitals, district general hospitals, private and community hospitals) and professional backgrounds (consultant gastroenterologists, colorectal surgeons, nurse endoscopists, screening practitioners, and senior trainees). Most participants were based in the UK, with a minority from other regions. Five endoscopists, chosen at random from those willing to repeat the assessment, comprised the intraobserver group. Although the cohort represents a broad range of clinical backgrounds, the predominance of UK-based participants and a relatively small intraobserver sample may affect generalizability.

### Study procedure

Participants completed the SMSA scoring online at their convenience. This digital format ensured uniform video quality and allowed asynchronous participation. The primary data collection phase involved scoring 10 unique polyp videos. To evaluate intraobserver reliability, five participants were randomly selected to repeat the assessment after a minimum of 2 weeks (up to 3 months). This interval was determined in consultation with a biostatistician to minimize recall bias. Participants were blinded to the fact that they were reassessing previously viewed videos.

We acknowledge that the intraobserver sample size (n = 5) is relatively small and may limit generalizability and statistical power of intraobserver agreement estimates. This sample size was determined based on feasibility during the COVID-19 pandemic and biostatistical consultation. The limited number may introduce variability and should be interpreted as preliminary data, highlighting the need for further studies with larger repeat-assessment groups.

### Data management and statistical planning

**Fig. 1:**
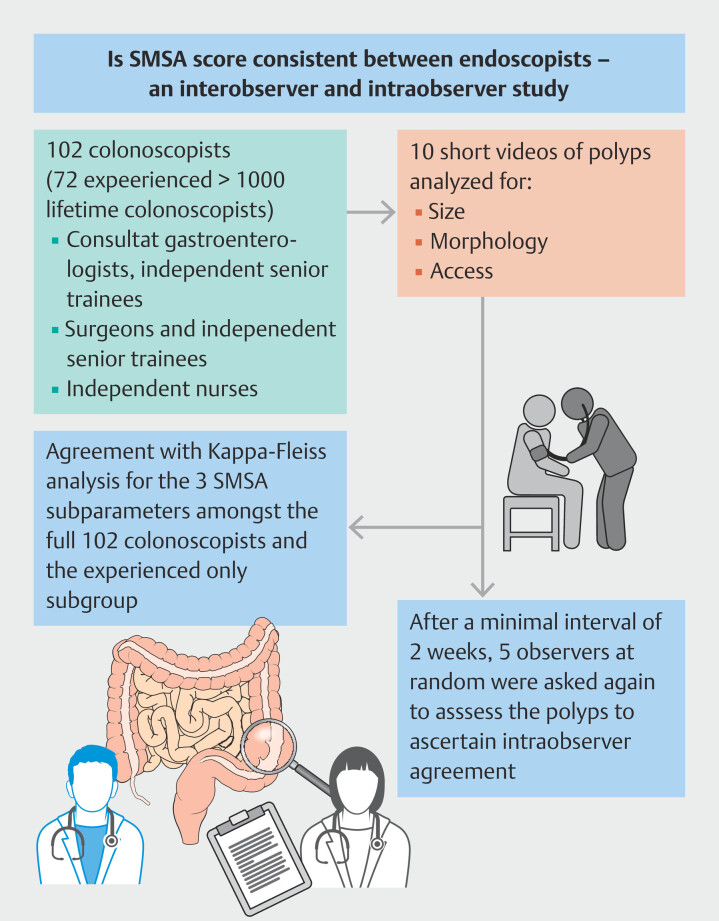
Methodical flowchart detailing consistency in assessment of 10 polyps, using SMSA score, among endoscopists with differing experiences.

All responses were captured digitally and compiled into structured datasets. The primary outcome was interobserver agreement for SMSA level across all raters. Secondary analyses included agreement on individual SMSA variables, subgroup analysis between experienced and less experienced endoscopists, and intraobserver consistency for repeat assessments. This dataset was later used for Fleiss’ kappa analysis and interpretation using the Landis and Koch classification. Fig. 1 outlines our methodical process.

### Outcomes assessment

The primary outcome of the study was to assess interobserver agreement among 102 independent endoscopists in assigning SMSA levels to 10 colonic polyps. SMSA scoring is a composite based on four components—size, morphology, site, and access. However, in this study, participants were asked to independently assess only three parameters—size, morphology, and access—whereas the site was fixed and provided alongside each video to reduce variability.

Therefore, in addition to evaluating overall SMSA level agreement, we also assessed individual agreement for size, morphology, and access because these reflect the subjective interpretation of each observer. Discrepancies or variations in these components could indicate aspects of the scoring system that are more prone to interpretation bias and which may benefit from clearer definitions or enhanced training.

Furthermore, intraobserver agreement was evaluated by inviting a subgroup of five participants to repeat the assessment of the same 10 polyps after an interval ranging from 2 weeks to 3 months. The purpose was to determine repeatability and internal consistency of SMSA scoring when conducted by the same observer over time. Discrepancies between the first and second assessments by the same individual were used to identify potential weaknesses in scoring reproducibility, particularly in parameters that rely heavily on estimation, such as polyp size and access.

### Statistical analysis

Fleiss’ kappa was used to assess agreement among multiple raters for categorical data because the SMSA score levels are ordinal but were analyzed as categorical variables. Kappa values and associated 95% confidence intervals were calculated using SPSS v26 (IBM), employing the standard error of kappa to generate confidence intervals. Statistical significance was assessed by comparing observed agreement to that expected by chance (null hypothesis: kappa = 0), and *P* < 0.05 was considered statistically significant. Although weighted kappa and intraclass correlation coefficients are suitable for some ordinal or continuous data, Fleiss’ kappa is recommended for agreement among more than two raters assessing categorical outcomes, which best fit our study design and SMSA score structure.

Initially, Fleiss’ kappa was calculated for the overall SMSA level assigned to each of the 10 polyps (by all 102 endoscopists) and for each of the three individual SMSA parameters (size, morphology, and access).

Subsequently, a subgroup analysis was performed to assess whether endoscopists with more procedure experience demonstrated improved agreement. For this, we selected the 72 participants with over 1,000 lifetime colonoscopies, categorizing them as the “experienced” group. Agreement levels for both overall SMSA scores and individual parameters were recalculated within this subgroup and compared with the general cohort.

For intraobserver agreement, kappa values were separately calculated for each of the five observers who completed the assessment twice. Size and SMSA level scores were compared across two time points.

Interpretation of kappa values followed the widely accepted classification proposed by Landis and Koch (1977), is outlined in Table 1 .

**Table:** **Table 1** Kappa value range and strength of agreement.

Kappa value range	Strength of agreement
< 0.00	Poor
0.00–0.20	Slight
0.21–0.40	Fair
0.41–0.60	Moderate
0.61–0.80	Substantial
0.81–1.00	Almost perfect

## Results

Table 2 presents participant characteristics of the 102 endoscopists who participated in the SMSA scoring study. The majority of participants were based in university hospital settings (67.6%), followed by district general hospitals (25.5%). Most respondents were consultant gastroenterologists (65.7%), with representation also from nurse endoscopists, colorectal surgeons, and senior trainees. In terms of experience, 74.5% had performed more than 1000 lifetime colonoscopies, qualifying them as experienced endoscopists. In addition, 35.3% of the cohort held British Colonoscopy Standards certification. Minor discrepancies in totals are due to a small number of incomplete responses in demographic fields.

**Table:** **Table 2** Participant characteristics of study participants (N = 102).

Variable	Category	Frequency (n)	Percentage (%)
Hospital setting	University hospital	69	67.6%
	District general hospital (DGH)	26	25.5%
	Private hospital	4	3.9%
	Community hospital	1	1.0%
	Total	100	98.0%*
Professional role	Consultant gastroenterologist	67	65.7%
	Nurse endoscopist	12	11.8%
	Colorectal surgeon	7	6.9%
	Screening practitioner	6	5.9%
	Independent trainee	8	7.8%
	Total	100	98.0%*
Endoscopy experience	< 500 lifetime colonoscopies	15	14.7%
	501–1000 procedures	9	8.8%
	> 1000 procedures	76	74.5%
	Total	100	98.0%*
BCS certification status	BCS certified	36	35.3%
	Not BCS certified	64	62.7%
	Total	100	98.0%*
*Note: Slight discrepancies in totals due to incomplete demographic responses from a few participants.			

Table 3 summarizes descriptive statistics for each of the 10 polyp cases included in the study. For every case, we present the mean, standard deviation, and 25th, 50th (median), and 75th percentiles for four SMSA-related variables: access, site, SMSA total score, and SMSA level. These statistics illustrate the central tendency and variability in how 102 endoscopists scored each polyp. Across the cases, the site parameter consistently showed no variability, with a standard deviation of 0 and fixed percentile values, because it was predefined and not subject to participant estimation. This uniformity confirms that participants interpreted this provided parameter consistently. Access, being one of the three estimated parameters, displayed moderate variability across the cases. For example, Case 1 had a relatively low mean access score (1.84 ± 0.99), whereas Cases 5 and 9 demonstrated higher mean access scores (2.84 ± 0.55 and 2.70 ± 0.72, respectively), reflecting differences in perceived complexity of scope positioning.

**Table:** **Table 3** SMSA rating for each case by endoscopists.

Case number	Category	Mean	SD	Percentiles		
				25th	50th	75th
Case 1	Access	1.84	0.992	1.00	1.00	3.00
	site	1.00	0.000	1.00	1.00	1.00
	SMSA	9.70	2.488	8.00	9.00	11.00
	level	2.63	0.800	2.00	2.00	3.00
Case 2	Access	2.48	0.882	1.00	3.00	3.00
	site	2.00	0.000	2.00	2.00	2.00
	SMSA	11.80	2.470	10.00	12.00	14.00
	level	3.21	0.640	3.00	3.00	4.00
Case 3	Access	2.66	0.755	3.00	3.00	3.00
	site	1.00	0.000	1.00	1.00	1.00
	SMSA	15.31	0.907	15.00	16.00	16.00
	level	4.00	0.000	4.00	4.00	4.00
Case 4	Access	1.30	0.718	1.00	1.00	1.00
	site	1.00	0.000	1.00	1.00	1.00
	SMSA	7.34	1.653	6.00	7.00	8.00
	level	2.13	0.367	2.00	2.00	2.00
Case 5	Access	2.84	0.545	3.00	3.00	3.00
	site	1.00	0.000	1.00	1.00	1.00
	SMSA	10.61	2.079	9.00	11.00	11.00
	level	2.86	0.779	2.00	3.00	3.00
Case 6	Access	2.06	1.003	1.00	3.00	3.00
	site	1.00	0.000	1.00	1.00	1.00
	SMSA	10.63	2.304	9.00	11.00	12.00
	level	2.84	0.762	2.00	3.00	3.00
Case 7	Access	1.04	0.281	1.00	1.00	1.00
	site	1.00	0.000	1.00	1.00	1.00
	SMSA	6.10	1.291	5.00	6.00	7.00
	level	1.67	0.493	1.00	2.00	2.00
Case 8	Access	1.12	0.477	1.00	1.00	1.00
	site	1.00	0.000	1.00	1.00	1.00
	SMSA	7.62	1.607	6.00	8.00	8.00
	level	2.14	0.403	2.00	2.00	2.00
Case 9	Access	2.70	0.718	3.00	3.00	3.00
	site	2.00	0.000	2.00	2.00	2.00
	SMSA	13.90	2.111	12.00	14.00	16.00
	level	3.69	0.545	3.00	4.00	4.00
Case 10	Access	2.32	0.952	1.00	3.00	3.00
	site	1.00	0.000	1.00	1.00	1.00
	SMSA	12.49	2.452	11.00	13.00	15.00
	level	3.50	0.718	3.00	4.00	4.00
SMSA, Size, Morphology, Site, and Access.						

SMSA total score varied widely between cases, indicating the diverse characteristics of the polyps chosen. For instance, Case 3 had the highest average SMSA score (15.31 ± 0.91), corresponding to a consistent assignment of SMSA level 4 (mean = 4.00, standard deviation = 0.00), suggesting a high-complexity lesion with little disagreement. In contrast, Case 7 had the lowest mean SMSA score (6.10 ± 1.29), aligning with a lower complexity polyp and a mean SMSA level of 1.67 (± 0.49), demonstrating more variability in perceived level. SMSA level, derived from the total SMSA score, also showed varying levels of agreement. Several cases (e.g., Cases 3 and 4) showed tight clustering around median values with low standard deviations, indicating consistent scoring by most participants. Others (e.g., Cases 1 and 10) showed more spread, suggesting greater variation in complexity assessment among endoscopists. These descriptive statistics provide insight into how subjective scoring influenced SMSA outcomes on a case-by-case basis and highlight specific cases with either strong consensus or variability among participants.

Table 4 presents results of interobserver agreement analysis for the SMSA scoring system across 10 different cases of colonic polyps, evaluated by multiple endoscopists. The Kappa values for the four parameters—access, site, SMSA, and level—ranged from fair to substantial agreement, with most parameters demonstrating fair to moderate agreement. Overall *P* values for each parameter were statistically significant ( *P* < 0.05), indicating that the observed agreements were not due to chance. Statistically significant kappa values indicate agreement beyond chance, but clinical relevance depends on the magnitude of kappa. For example, “fair” agreement may not be sufficient for reliable clinical use, underscoring the need for improvement in SMSA reproducibility despite statistically significant results.

**Table:** **Table 4** SMSA agreement levels for each case.

Case number	Category	K(SE)	Agreement	95%CI (lower)	95% CI (upper)	Significance
Case 1	access	0.39 (0.32)	Fair	0.32	1.39	0.029
	site	0.35 (0.37)	Fair	0.33	1.51	0.041
	SMSA	0.36 (0.35)	Fair	0.34	1.41	0.014
	level	0.39 (0.27)	Fair	0.34	1.22	0.031
Case 2	Access	0.56 (0.18)	Moderate	0.54	1.21	0.042
	site	0.36 (0.20)	Fair	0.31	1.18	0.041
	SMSA	0.42 (0.25)	Fair	0.34	1.18	0.033
	level	0.44 (0.14)	Fair	0.21	1.32	0.032
Case 3	Access	0.37 (0.37)	Fair	0.24	1.38	0.018
	site	0.45 (0.15)	Moderate	0.21	1.1	0.036
	SMSA	0.42 (0.31)	Fair	0.36	1.55	0.021
	level	0.45 (0.32)	Moderate	0.35	1.37	0.012
Case 4	Access	0.37 (0.17)	Fair	0.23	1.41	0.013
	site	0.41 (0.17)	Moderate	0.27	1.25	0.048
	SMSA	0.6 (0.24)	Moderate	0.41	1.31	0.03
	level	0.62 (0.23)	Moderate	0.51	1.21	0.014
Case 5	Access	0.36 (0.48)	Fair	0.25	1.59	0.017
	site	0.42 (0.25)	Moderate	0.26	1.45	0.03
	SMSA	0.21 (0.24)	Fair	0.18	1.46	0.044
	level	0.57 (0.13)	Moderate	0.46	1.1	0.027
Case 6	Access	0.19 (0.14)	Fair	0.66	1.11	0.018
	site	0.44 (0.14)	Moderate	0.37	1.28	0.042
	SMSA	0.38 (0.32)	Fair	0.27	1.58	0.032
	level	0.58 (0.22)	Moderate	0.46	1.19	0.015
Case 7	Access	0.64 (0.41)	Substantial	0.49	1.62	0.023
	site	0.38 (0.09)	Fair	0.18	1.16	0.034
	SMSA	0.19 (0.16)	Fair	0.14	1.4	0.048
	level	0.72 (0.20)	Substantial	0.67	1.26	0.018
Case 8	Access	0.36 (0.34)	Fair	0.25	1.37	0.015
	site	0.42 (0.22)	Fair	0.33	1.47	0.018
	SMSA	0.6 (0.38)	Moderate	0.59	1.58	0.034
	level	0.18 (0.22)	Fair	0.14	1.31	0.032
Case 9	Access	0.37 (0.23)	Fair	0.34	1.14	0.032
	site	0.41 (0.20)	Fair	0.32	1.47	0.034
	SMSA	0.62 (0.35)	Moderate	0.54	1.59	0.042
	level	0.23 (0.12)	Fair	0.12	1.24	0.022
Case 10	Access	0.38 (0.20)	Moderate	0.32	1.35	0.048
	site	0.73 (0.28)	Substantial	0.62	1.47	0.043
	SMSA	0.42 (0.27)	Fair	0.31	1.29	0.027
	level	0.4 (0.35)	Fair	0.39	1.48	0.037
SMSA, Size, Morphology, Site, and Access.						

Table 5 presents interobserver agreement for the SMSA scoring system, assessed across multiple domains and stratified by parameter and subcategories. Agreement was evaluated using Fleiss’ kappa (κ) for the following key SMSA components: size, morphology, access, and SMSA level, along with their relevant subcategories. Among the primary SMSA parameters, the highest agreement was observed for morphology (κ = 0.505), interpreted as moderate agreement, followed by access (κ = 0.408, moderate) and SMSA level (κ = 0.346, fair). The size parameter yielded the lowest agreement (κ = 0.241, fair), reflecting the challenge of consistently estimating polyp size visually.

**Table:** **Table 5** SMSA interobserver agreement.

Parameter	K(SE)	Agreement	95%CI (lower)	95%CI (upper)	Significance
Size	0.241(0.002)	Fair	0.236	0.246	*P <* 0.001
Morphology	0.505(0.003)	Moderate	0.498	0.512	*P <* 0.001
Access	0.408(0.004)	Moderate	0.399	0.417	*P <* 0.001
SMSA level	0.346(0.003)	Fair	0.34	0.351	*P <* 0.001
Size range (POINTS)					
< 1 cm (1)	0.207(0.006)	Fair	0.195	0.219	*P <* 0.001
1.0–1.9 cm (3)	0.219(0.006)	Fair	0.207	0.231	*P <* 0.001
2.0–2.9 cm (5)	0.159(0.006)	Slight	0.147	0.171	*P <* 0.001
3.0–3.9 cm (7)	0.148(0.006)	Slight	0.134	0.16	*P <* 0.001
> 4.0 cm (9)	0.564(0.006)	Moderate	0.552	0.576	*P <* 0.001
Morphology					
Pedunculated	0.657(0.006)	Substantial	0.645	0.669	*P <* 0.001
Sessile	0.524(0.006)	Moderate	0.511	0.536	*P <* 0.001
Flat	0.46(0.006)	Moderate	0.448	0.472	*P <* 0.001
SMSA, Size, Morphology, Site, and Access.					

Further analysis by size subcategories revealed that interobserver agreement varied significantly across ranges. The highest kappa value was seen in the > 4.0 cm category (κ = 0.564, moderate), likely due to the broader range and distinctiveness of large polyps. In contrast, smaller size categories—such as 2.0–2.9 cm (κ = 0.159) and 3.0–3.9 cm (κ = 0.148)—showed only slight agreement, indicating substantial variability among endoscopists in estimating mid-range polyp sizes. Morphological subcategories demonstrated stronger reliability. The pedunculated category showed the highest agreement (κ = 0.657, substantial), followed by sessile (κ = 0.524) and flat (κ = 0.460), both of which were classified as moderate agreement. These findings suggest that visual identification of polyp morphology may be more consistent than size estimation across observers.

All observed kappa values in Table 5 were statistically significant ( *P* < 0.001), indicating that the agreements were unlikely due to chance. The overall results underscore variability in scoring certain parameters and point to size estimation as a potential weakness in SMSA system reproducibility.

The kappa value ranged from moderate agreement (0.444) to perfect agreement (1.000) with respect to size assessment and ranged from moderate agreement (0.429) to almost perfect (0.846) with respect to SMSA assessment. For any given observer, size agreement appeared to surpass SMSA agreement. Three of the five observers demonstrated similar agreement categories between size and SMSA. Also, through observation, it can be noted that as the size agreement increases, so does the agreement for SMSA ( Table 6 ).

**Table:** **Table 6** Intraobserver agreement.

Observer	Agreement value in Kappa (Size)	Agreement value in Kappa (SMSA Level)	Significant
1	0.747	0.595	0.001
2	0.444	0.429	0.014
3	0.839	0.643	0.006
4	1	0.846	< 0.001
5	0.737	0.706	< 0.001
SMSA, Size, Morphology, Site, and Access.			

## Discussion

The European Society of Gastrointestinal Endoscopy recommends SMSA scoring as part of pre-resection planning for large or complex colorectal lesions, recognizing its value in risk stratification and procedure planning 8 13 . Our findings affirm that, although SMSA provides a useful framework, there is significant variation in how endoscopists interpret and apply its individual components—particularly among clinicians with different levels of experience and exposure to complex polypectomy.

In our study, morphology had the highest interobserver agreement, especially for clearly defined categories such as pedunculated, consistent with prior observations 14 . Conversely, access scored lowest, likely due to video limitations that omit tactile or dynamic factors such as torque, looping, or patient positioning—factors crucial in real-time assessments. These findings reinforce that static imaging or video clips may not fully capture the procedure nuance necessary for accurate access grading.

Size, although numerically dominant within SMSA, emerged as another key source of disagreement, particularly in the intermediate subcategories (e.g., 2–2.9 cm and 3–3.9 cm). As a continuous variable segmented into five categories, its interpretation appears inherently subjective, even with visual cues. Collapsing size categories into broader ranges—such as < 2 cm, 2–3.9 cm, and ≥ 4 cm—may enhance reproducibility, because our data suggest better agreement at the extremes of the scale. These proposals align with similar calls in the literature to simplify scoring frameworks in endoscopic complexity tools 7 14 .

Importantly, observers who completed both rounds showed stronger intraobserver agreement on size, suggesting memory or internal calibration effects. Moreover, endoscopists with greater experience or CRC screening exposure displayed higher consistency across all domains, echoing other studies that emphasize the role of case volume and specialty focus in diagnostic accuracy 10 15 .

With the increasing availability of AI-assisted systems that support polyp classification and sizing in real time 11 12 , the relevance of SMSA must be reevaluated. Although such technologies show promise in reducing variability and improving decision-making, they are not yet widely adopted or validated for complexity scoring. Until such tools become standard, structured training and calibration exercises in SMSA scoring remain critical, especially in multidisciplinary teams and training environments.

Our study's limitations include a modest number of observers due to the specialized nature of the task and the constraints imposed by the COVID-19 pandemic, which restricted recruitment and collaboration. Nevertheless, these findings underscore the need for refinement of complexity scoring tools and highlight opportunities for digital augmentation and targeted education. Furthermore, another limitation of our study is the small intraobserver sample size, which restricts statistical confidence and the ability to generalize intraobserver reliability results. Future studies should include larger intraobserver samples to confirm these findings.

Our study also is limited by the relatively small number of polyp video cases (n=10), which may not capture the full spectrum of polyp morphology, size, and complexity encountered in clinical practice. Larger studies with a broader selection of polyp cases are needed to further validate our findings.

In addition, the majority of participating endoscopists were based in the UK (82/102, 80.4%), which may introduce regional bias and limit generalizability of the results to countries or healthcare systems with different endoscopic training standards. Future multicenter studies with broader international representation are warranted.

Our study focused on interobserver and intraobserver variability in SMSA scoring but did not examine how such variability may affect clinical outcomes such as complication rates, completeness of resection, or referral accuracy. Further research is required to investigate clinical implications of observer variation in SMSA application.

## Conclusions

This study highlights significant interobserver and intraobserver variability in application of the SMSA scoring system, particularly within the domains of size and access. Although morphology demonstrated relatively high agreement—especially in well-defined categories such as pedunculated lesions—size assessment emerged as the most inconsistent, likely due to its continuous nature and subjective visual estimation. Our findings suggest that consolidation of SMSA size subcategories may improve reproducibility and scoring reliability. Furthermore, the role of observer experience and training background appears central to consistency of scoring, underscoring the need for standardized education and calibration exercises.

Despite its widespread adoption, SMSA has limitations in the era of rapidly evolving technologies. The rise of adjunctive tools such as AI, CADx, and enhanced imaging modalities offers an opportunity to reduce subjectivity and improve precision of polyp characterization. These tools may enhance accuracy of pre-resection planning and triage, especially for complex lesions.

### Future directions

Future research should aim to refine the SMSA system by exploring a simplified scoring matrix—particularly for the size and access domains—to improve interrater agreement. Validation studies with larger, more diverse cohorts of endoscopists, stratified by experience and subspecialty exposure, are essential. In addition, prospective studies incorporating AI-assisted polyp characterization and telestration platforms could assess their potential in reducing observer variability and enhancing scoring accuracy.

There is also a pressing need to integrate SMSA scoring into electronic health records and referral systems with automated decision support, reducing manual errors and standardizing risk assessment at the point of care. Finally, future iterations of SMSA might benefit from combining endoscopic parameters with patient-specific factors and histological predictors to develop a more holistic, risk-adjusted approach to complex polyp management.

## Data availability statement

The datasets generated and/or analyzed during the current study are available from the corresponding author on reasonable request, subject to institutional data sharing policies and ethical approval requirements.
